# Short term effects of coffee components consumption on gut microbiota in patients with non-alcoholic fatty liver and diabetes: A pilot randomized placebo-controlled, clinical trial

**DOI:** 10.17179/excli2019-2021

**Published:** 2020-03-02

**Authors:** Asieh Mansour, Mohammad Reza Mohajeri-Tehrani, Sara Karimi, Milad Sanginabadi, Hossein Poustchi, Samaneh Enayati, Saeedeh Asgarbeik, Javad Nasrollahzadeh, Azita Hekmatdoost

**Affiliations:** 1 Department of Clinical Nutrition and Dietetics, Faculty of Nutrition and Food Technology, National Nutrition and Food Technology, Research Institute Shahid Beheshti University of Medical Science, Tehran, Iran; 2 Endocrinology and Metabolism Research Center, Endocrinology and Metabolism Clinical Sciences Institute, Tehran University of Medical Sciences, Tehran, Iran; 3 Radiology Department, Shariati Hospital, Tehran University of Medical Sciences, Tehran, Iran; 4 Liver and Pancreatobiliary Diseases Research Center, Digestive Diseases Research Institute, Tehran University of Medical Sciences, Tehran, Iran; 5 Metabolic Disorders Research Center, Endocrinology and Metabolism Molecular Cellular Sciences Institute, Tehran University of Medical Sciences, Tehran, Iran

**Keywords:** coffee, caffeine, chlorogenic acid, microbiota, fatty liver, diabetes, clinical trial

## Abstract

The aim of this study was to determine the effects of caffeine and chlorogenic acid supplementation on gut microbiota, and metabolic disturbances in patients with NAFLD and diabetes. In this randomized, placebo-controlled, clinical trial, 26 patients with diabetes and NAFLD were randomly assigned to four groups to receive either 200 mg caffeine plus 200 mg chlorogenic acid (CFCA), or 200 mg caffeine plus 200 mg placebo (starch) (CFPL), or 200 mg chlorogenic acid plus 200 mg placebo (CAPL), or 200 mg placebo plus 200 mg placebo (PLPL) for 12 weeks. After 3 months of supplementation, patients in the intervention groups showed a significant decrease in body weight (CFCA group =-3.69 kg; CFPL group=-0.7kg; CAPL group=-0.43kg; PLPL group=0.26 kg) (p=0.004). Weight reduced significantly more in CFCA group compared to all other three groups (p=0.005 for PLPL; p=0.023 for CAPL; and p=0.031 for CFPL). Although the number of gut Bifidobacteria increased in CFCA group, there were no statistically significant differences within and between the groups in any of bacteria numbers. In conclusion, our study showed that 12 weeks consumption of 200 mg/day caffeine plus 200 mg/day chlorogenic acid is effective in reduction of weight in patients with NAFLD and diabetes which might be at least partially through the rise in gut Bifidobacteria. This pilot study shed a light on the pathway of future clinical trials assessing the effects of coffee consumption in these patients. This trial has been registered at clinicaltrial.gov with registration number of NCT02929901.

## Introduction

Coffee is the most popular drink worldwide. Thus, its impact on human body could be important on a population scale. Coffee is produced from the coffee plant seeds, genus Coffee, which contains over 1000 bioactive compounds (Gomez-Juaristi et al., 2018[11]; Jeszka-Skowron et al., 2015[16]). The most important bioactive compounds of coffee are chlorogenic acids, and caffeine; however, the biochemical compositions of the final coffee are various, based on the type of seeds, and methods of preparation.

The association between coffee consumption and all-cause and cause-specific mortality has been investigated in several observational studies. Most of these studies have reported an inverse association between all-cause and cause-specific mortality and both caffeinated and decaffeinated coffee consumption (Grosso et al., 2016[12]). Moreover, the negative association of coffee consumption with metabolic, gastrointestinal, and liver disorders have been reported previously (Awaad et al., 2011[3]; Gutierrez-Grobe et al., 2012[13]; Hodge et al., 2017[14]; Kennedy et al., 2016[19]; Molloy et al., 2012[24]; Santos and Lima, 2016[31]; Vitaglione et al., 2012[34]). A recent umbrella review and meta-analysis on the association between coffee consumption and health concluded that the beneficial associations between coffee consumption and liver outcomes have relatively large and consistent effect sizes compared with other outcomes (Poole et al., 2017[28]). 

There are several limitations in previous studies evaluating the relationship between coffee consumption and chronic disorders. Firstly, most of these studies were observational investigations. Thus, they could not make causative claims. Secondly, there are several confounding variables in determination of exposure including coffee composition, methods of preparation, and irregularity in coffee consumption habits. 

All of these limitations suggest the necessity of conducting controlled clinical trials with known amounts of coffee components to find any possible cause-effect relationship, while addressing the mechanism of action. On the other hand, a recent study on 16 healthy subjects has reported that 3 cups/day coffee consumption for 3 weeks increased numbers of the Bifidobacterium spp. population in fecal samples of participants (Jaquet et al., 2009[15]). Thus, we presumed that the beneficial associations of coffee consumption with metabolic disorders might be due to its effects on gut microbiota; however, the probable acting component of it is not known. Therefore, we designed this pilot study to assess the effects of two main components of coffee, caffeine and chlorogenic acid, on gut microbiota in patients with non-alcoholic fatty liver disease (NAFLD) and diabetes type 2.

## Material and Methods

### Recruitment and eligibility screening

Patients aged 30-65 years old with a history of diabetes were identified and recruited from an Endocrinology clinic at Shariati hospital, Tehran, Iran. The diagnosis of NAFLD was made by the presence of steatosis on ultrasound examination (US), associated with steatosis of grade 2 or more in the Fibroscan exam. Exclusion criteria were consumption of any kind of antibiotics 2 weeks before and during the study, being professional athlete, taking any medications other than oral diabetes drugs, viral hepatitis, alcohol use, hepatic cirrhosis, other causes of chronic liver disease, hypothyroidism, renal, intestinal, and cardiovascular disorders, body mass index (BMI)>35 kg/m^2^, being on a special diet, any change in hypoglycemic medications, psychiatric disorders impairing the patient's ability to provide written informed consent, as well as pregnancy, lactation. 

### Study design

All eligible patients with diabetes type 2 were recruited. After explanation of the study protocol, patients signed an informed consent form, which was approved by the ethics committee of the National Institute of Medical Research Development (NIMAD). The patients were undertaken a US exam. If there was any evidence of hepatic steatosis of grade 2 or more in US exam, they were undertaken a Fibroscan test. Those patients who had steatosis of grade 2 or more in Fibroscan exam (Controlled attenuation parameter (CAP) score >263) were enrolled in this study. 

The enrolled participants were randomly assigned to four groups to receive either 200 mg caffeine plus 200 mg chlorogenic acid (CFCA), or 200 mg caffeine plus 200 mg placebo (starch) (CFPL), or 200 mg chlorogenic acid plus 200 mg placebo (CAPL), or 200 mg placebo plus 200 mg placebo (PLPL) for 12 weeks. Randomization lists were computer-generated by a statistician and given to the interviewer. Subjects, investigators, and staff were blinded to the treatment assignment until the end of the study. At the time of randomization, baseline data were collected and participants were provided with a 4-week supply of supplements. Participants were followed up every 4 weeks to assess their compliance. Adherence was assessed with capsule counts confirmed at each visit. All supplements were provided by Arjuna Natural Extract, India. 

### Clinical, paraclinical, and dietary intake assessment

Anthropometric measurements were done for all participants at the beginning of the study and at the 12^th^ week of intervention. Body mass index (BMI) was calculated by dividing the patient's weight in kilogram by his or her height in meters squared. Waist circumstance was measured according to WHO recommendation (WHO, 2012[36]). 

At the beginning and the end of study, fasting blood, and stool sample were collected, and were stored at -80 °C freezer until the analysis. All biochemical measurements were done at the same lab, by the same technician, using standard methods. The fasting glucose concentration was assessed using the GOD/POD method. Fasting insulin concentration was assessed by an Enzyme-linked immunosorbent assay (Mercodia AB, Sweden). The homeostasis model assessment - insulin resistance (HOMA-IR) was used to determine the degree of insulin resistance using the following formula (Matthews et al., 1985[21]): HOMA-IR = [fasting insulin (mU/L) × fasting blood glucose (mg/dL)]/405. γ-glutamyltransferase (GGT) was measured using enzymatic colorimetric assay (Parsazmoun, Tehran, Iran). Alanine aminotransferase (ALT), and aspartate aminotransferase (AST) concentrations were measured using photometric assay (Reckon, Vadodara, India). High sensitive-C reactive protein (hs-CRP) concentration was measured using an Enzyme-linked immunosorbent assay (Diagnostics Biochem Canada Inc.). 

To assess nutrition intake, dietary food records for three days including a weekend day were collected at the beginning and the end of study. Dietary intakes were then analyzed using Nutritionist 4 (First DataBank, San Bruno, CA), incorporating the use of food scales and models to enhance portion size accuracy. Physical activity was also assessed using the Metabolic Equivalent of Task (MET) questionnaire (Ainsworth et al., 2000[2]) at the beginning and the end of this study.

Resting energy expenditure (REE) was measured at the beginning and the end of study using FitMate™ metabolic analyzer (Cosmed, Rome, Italy). The details of procedure were described previously (Shaneshin et al., 2011[32]). Body composition was assessed using body impedance analyzer (BIA) (Tanita, Ilinois, USA) at the beginning and the end of study.

### Intestinal microbial analysis

Fecal samples were collected in sterile containers at the beginning and end of the study. The samples were brought to the laboratory frozen, and were stored at -80 °C until analyzed. 

DNA extraction from 1 gram of stools was done by using the QIAamp DNA Stool Mini Kit (Qiagen) according to the manufacturer's instructions. 

Reverse-transcriptase polymerase chain reaction (RT-qPCR) analysis was performed using a Light Cycler 480 system (Roche, Germany). The group and species-specific primers for PCR are listed in Table 1(Tab. 1) (Yoon et al., 2014[37]). The primers were synthesized commercially by Takapouzist (Tehran, Iran). Quantitative PCR was performed in 96-well plates in final volumes of 20 μl consisting of 1 μl of fecal DNA, 0.5 μl of primers (10 pmol each), 10 μl SYBR Green I master (Roche, Germany), and 8 μl of H_2_O. PCR amplification involved: pre-incubation at 94 °C for 4 min followed by 55 cycles of amplification (denaturation at 94 °C for 15 sec, primer annealing at 55 °C for 15 sec, and elongation at 72 °C for 20 sec). Melting curves were obtained by heating samples from 50 °C to 90 °C at a rate of 5 °C/s.

### Statistical analysis

Analysis was conducted using SPSS 20.0 (for Windows SPSS Inc., Chicago, IL). Data for normally distributed and categorical variables are reported as mean ± standard deviation and frequency percentage, respectively. An analysis of variance (ANOVA) was used to assess the differences among groups at baseline. Comparisons of changes (endpoint minus baseline) after intervention between groups were done using ANOVA, while using Post Hoc Tukey test to compare groups' changes. To control confounding variables, analysis of covariance (ANCOVA) test was applied. ANCOVA analysis was adjusted for the baseline value of each variable, dietary intake of energy, coffee, tea, and fibre. Statistical significance was set at p<0.05, based on two-sided tests. 

## Results

**Figure 1:**
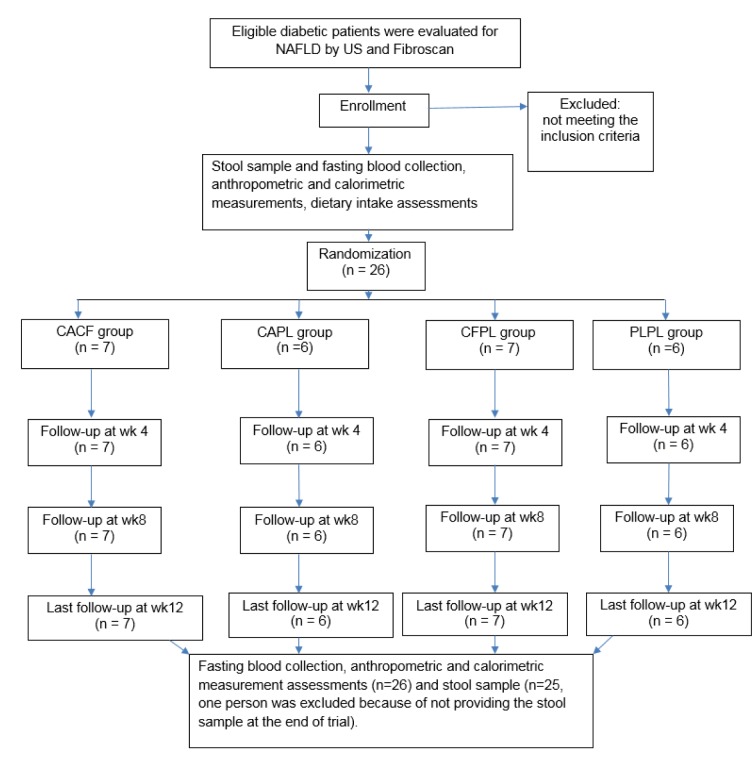
Study consort flow chart CF, Chlorogenic acid; CA, caffeine; PL, placebo

The study consort flow chart is depicted in Figure 1(Fig. 1). None of the participants were excluded from the study during 12 weeks of follow-up. All participants consumed more than 98 % of their supplements according to the capsule counts in each follow-up visit. Baseline characteristics of the patients across the four groups are presented in Table 2(Tab. 2). As it is shown, all trial arms were well matched in respect to baseline characteristics. Table 3(Tab. 3) shows the effects of each supplementation on anthropometric and biochemical measurements after 12 weeks. Weight reduced significantly more in CFCA group compared to all other three groups (p=0.005 for PLPL; p=0.023 for CAPL; and p=0.031 for CFPL). Moreover, BMI decreased in CFCA significantly more than PLPL, and CAPL groups (p=0.009, and p=0.033, respectively). Furthermore, energy intake declined in CFPL group significantly more than PLPL group (p=0.032). There were no other significant differences in changes of biochemical and anthropometric measurements among the four groups. In addition, dietary intakes of fibres, tea, coffee, macronutrients, and micronutrients were not significantly different between and within the groups. 

**Figure 2:**
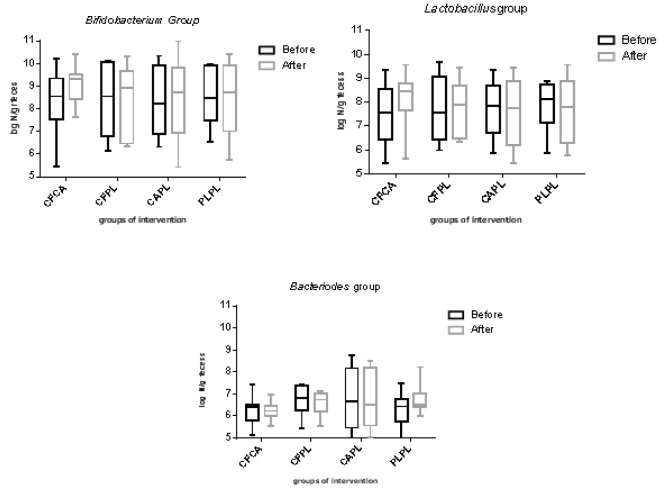
The log_10 _number per gram of feces of bacteria in each group CF, Chlorogenic acid; CA, caffeine; PL, placebo

The log_10_ number of Bifidobacteria, Lactobacillus, and Bacteriodes bacteria per one gram of feces before and after intervention in each group is demonstrated in Figure 2(Fig. 2). Although the number of gut Bifidobacteria increased in CFCA group, there were no statistically significant differences within and between the groups in any of bacteria numbers. No adverse effects were reported during the study period in any participant.

For more results see the Supplementary data.

## Discussion

This pilot randomized, placebo-controlled, clinical trial has shown that 200 mg/day caffeine plus 200 mg/day chlorogenic acids supplementation for 12 weeks was effective in weight reduction in patients with diabetes and NAFLD, probably due to the rise in the number of gut Bifidobacteria.

Previous observational studies have shown the inverse association between coffee consumption and risk of NAFLD (Catalano et al., 2010[5]; Chen et al., 2014[6], 2019[7]), and diabetes (Santos and Lima, 2016[31]). However, the results of these studies are, by their nature, open to dispute because they run the risk of containing confounding biases. Thus, experimental studies were conducted to find a possible cause-effect mechanism. Although the results of the experimental studies are inconsistent, they have reported promising effects from either coffee, or its main constitutes (caffeine and chlorogenic acids) (Salomone et al., 2017[30]). Some of these studies have reported that coffee and chlorogenic acid can modify gut microbiota (Caro-Gomez et al., 2019[4]; Cowan et al., 2014[8]; Pan et al., 2016[25]); however, the results were not similar. We found only one human study that evaluated the effects of coffee consumption on human gut microflora (Jaquet et al., 2009[15]). Although the result of this study was not consistent with the experimental studies, it is in line with our results. Jaquet et al. (2009[15]) have reported that 3 weeks consumption of 3 cups/day coffee only increased Bifidobacterium spp, without any effects on other dominant microbiota bacteria. We also observed that the patients on caffeine plus chlorogenic acid (CFCA) supplementation had a rise in number of Bifidobacterium bacteria; however, this rise was not statistically significant. Although it seems that the statistical meaningful of a rise in bacteria might not be a predictor of clinical effects of it, we presume that this increment did not reach to a statistical significant level due to some methodological differences between our study and the previous study by Jaquet et al. Firstly, we used only two main components of coffee, while jaquet et al used the whole coffee as the intervention of the study. So other components of coffee might have affected the gut microbiota (Cuervo et al., 2016[9]; Gniechwitz et al., 2007[10]; Perez-Burillo et al., 2019[27]; Vaughan et al., 2015[33]). Secondly, as a pilot study, we used the minimum dosage of supplementation, equal to 2 cups coffee/day, to avoid the possible side effects of the supplements, while Jaquet et al. used the dosage of 3 cups/day. Thus, it is possible that our dosage was not as high as exploring a statistically significant increment in Bifidobacteria group. Finally, Jaquet et al. have reported the rise only in Bifidobacterium spp. subgroup, whereas we measured the whole Bifidobacterium bacteria group. It might be possible that we could find a statistical significant difference in subgroups of Bifidobacteria. Therefore, future studies are needed to assess whole gut microbiota to find the possible alterations in bacteria subgroups. 

Supplementation with both caffeine and chlorogenic acid (CFCA) had some beneficial effects on metabolic disturbances of participants; however, the effect was statistically significant only in the case of weight loss. Previous experimental studies have also reported the effects of coffee consumption on weight loss (Salomone et al., 2017[30]), but human studies are limited to healthy volunteers without assessment of weight loss (Wedick et al., 2011[35]), and with controversial results in assessment of other metabolic disturbances (Agudelo-Ochoa et al., 2016[1]; Kempf et al., 2010[18]; Lecoultre et al., 2014[20]; Moisey et al., 2008[22]; Papakonstantinou et al., 2016[26]). In our study, the significant weight reduction might be due to increase in the gut Bifidobacteria (Karbaschian et al., 2018[17]; Mokhtari et al., 2019[23]; Rashvand et al., 2018[29]). Whether the dosage of our supplements was not enough to induce a significant effect on metabolic disturbances, or other components of coffee might be effective to get the beneficial results, remained to be elucidated in future studies.

The most important advantage of this study is that it is the first randomized, placebo-controlled, clinical trial on patients with NAFLD and diabetes evaluating the effects of two main components of coffee on gut microbiota. Since it was a pilot study, it had some limitations such as low sample size, short study duration, and limitation in supplements dosage, and number of measured bacteria; however, it could shed a light on the future pathway of clinical trials to assess the effects of coffee and its components in patients with NAFLD and/or diabetes.

## Conclusion

In conclusion, our study showed that 12 weeks consumption of 200 mg/day caffeine plus 200 mg/day chlorogenic acid is effective in reduction of weight in patients with NAFLD and diabetes without any side effects. These effects might be at least partially through the rise in gut Bifidobacteria. This pilot study provided a clue for future clinical trials in these patients.

## Abbreviations

NAFLD, Non-alcoholic fatty liver disease; WC, waist circumference; BMI, body mass index; MET, metabolic equivalent of tasks; ALT, alanin aminotransferase; AST, aspartate aminotransferase; GGT, γ-glutamyl transferase; FBS, fasting blood sugar; LDL-C, low density lipoprotein; HDL-C, high density lipoprotein; hs-CRP, high sensitive C reactive protein; REE, resting energy expenditure; CF, Caffeine; CA, chlorogenic acid; PL, placebo.

## Acknowledgements

The study was supported by National Institute for Medical Research Development (NIMAD) to AH with grant number of 963356.

## Authors’ contributions

AM, MRM, and AH developed the proposal, obtained ethical approvals, applied for funding, supervised data collection and prepared the first draft. AM and AH conceived the idea, provided expertise in designing and analysis of the study. SK, MS, HP, SE, and SSA were involved in study experiments and analysis.

## Ethics approval and consent to participate

Ethical clearance was taken from the National Institute for Medical Research Development (NIMAD) with number of IR-NIMAD-REC.1396.191. Informed consent was taken from all participants.

## Competing interests

The authors declare that they have no competing interests.

## Supplementary Material

Supplementary data

## Figures and Tables

**Table 1:**
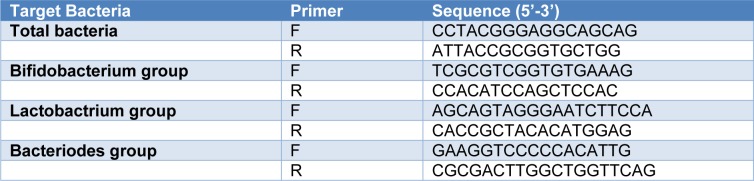
Primer sequences used for quantitative real-time PCR assays for each bacteria

**Table 2:**
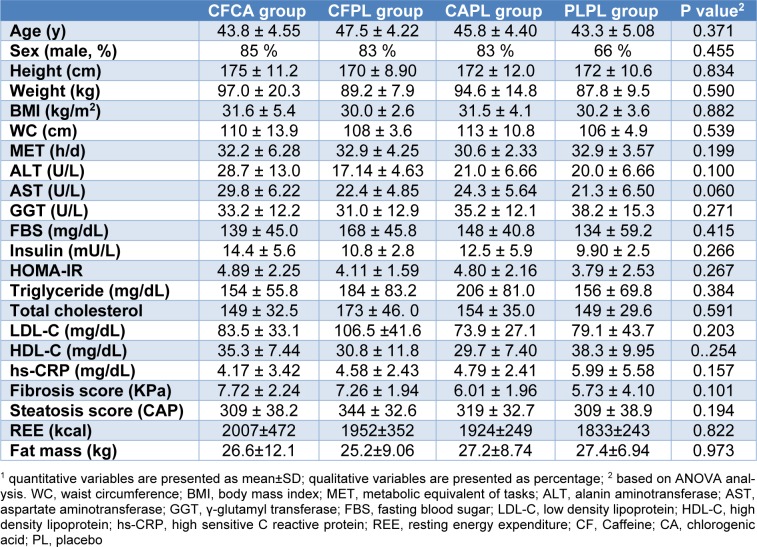
Baseline characteristics at enrollment^1^

**Table 3:**
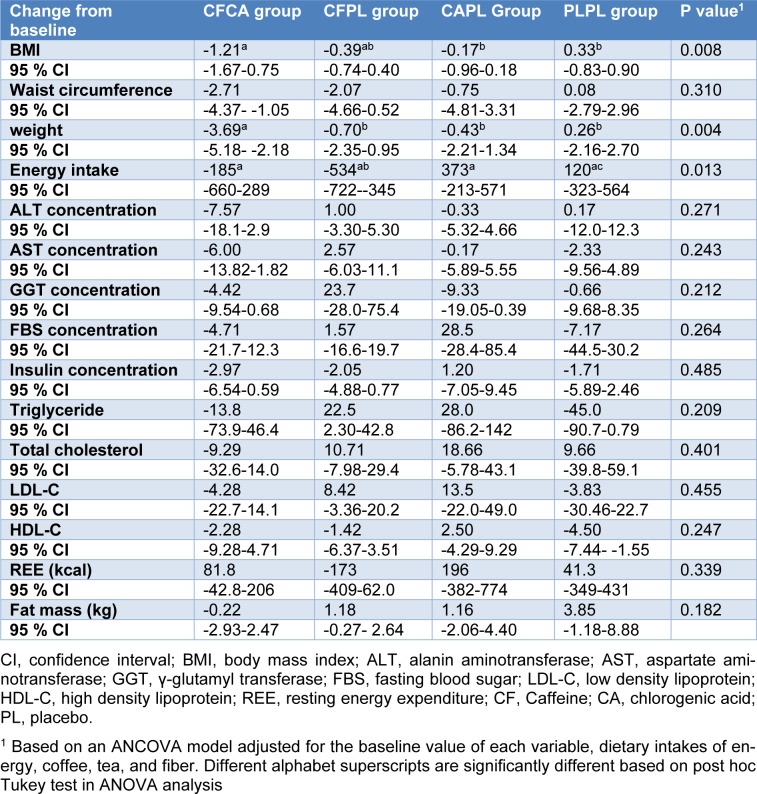
Mean changes (95 % CI) during study across 4 studied groups
