# Supplementary material for: Short term effects of coffee components consumption on gut microbiota in patients with non-alcoholic fatty liver and diabetes: A pilot randomized placebo-controlled, clinical trial
Source: EXCLI J. 2020 Mar 2;19:241–50. doi: 10.17179/excli2019-2021 (PMC7105939; doi:10.17179/excli2019-2021)
Supplement: Supplementary data [file EXCLI-19-241-s-001.pdf]

EXCLI Journal 2020;19:241-250 – ISSN 1611-2156
Received: November 30, 2019, accepted: February 24, 2020, published: March 02, 2020

Supplementary data to:

SHORT TERM EFFECTS OF COFFEE COMPONENTS CONSUMPTION ON GUT MICROBIOTA IN PATIENTS WITH NON-ALCOHOLIC FATTY LIVER AND DIABETES: A PILOT RANDOMIZED PLACEBO-CONTROLLED, CLINICAL TRIAL

Asieh Mansour[1,2], Mohammad Reza Mohajeri-Tehrani[2], Sara Karimi[1], Milad Sanginabadi[3], Hossein Poustchi[4], Samaneh Enayati[5], Saeedeh Asgarbeik[5], Javad Nasrollahzadeh[1], Azita Hekmatdoost[1*]

[1] Department of Clinical Nutrition and Dietetics, Faculty of Nutrition and Food Technology, National Nutrition and Food Technology, Research Institute Shahid Beheshti University of Medical Science, Tehran, Iran
[2] Endocrinology and Metabolism Research Center, Endocrinology and Metabolism Clinical Sciences Institute, Tehran University of Medical Sciences, Tehran, Iran
[3] Radiology Department, Shariati Hospital, Tehran University of Medical Sciences, Tehran, Iran
[4] Liver and Pancreatobiliary Diseases Research Center, Digestive Diseases Research Institute, Tehran University of Medical Sciences, Tehran, Iran
[5] Metabolic Disorders Research Center, Endocrinology and Metabolism Molecular Cellular Sciences Institute, Tehran University of Medical Sciences, Tehran, Iran

* **Corresponding author**: Azita Hekmatdoost, Department of Clinical Nutrition and Dietetics, Faculty of Nutrition and Food Technology, National Nutrition and Food Technology, Research Institute Shahid Beheshti University of Medical Science, Tehran, Iran, E-mail: a_hekmat2000@yahoo.com

http://dx.doi.org/10.17179/excli2019-2021

This is an Open Access article distributed under the terms of the Creative Commons Attribution License (http://creativecommons.org/licenses/by/4.0/).

EXCLI Journal 2020;19:241-250 – ISSN 1611-2156
Received: November 30, 2019, accepted: February 24, 2020, published: March 02, 2020

Supplementary Table 1: Raw data of the anthropometric parameters, REE and physical activity in each group before and after intervention. The below raw data are related to Table 1 and 2 in the main text.

Code	Weight (kg) Before	Weight (kg) After	BMI (kg/m^2) Before	BMI (kg/m^2) After	Fat mass(kg) Before	Fat mass(kg) After	Waist (cm) Before	Waist (cm) After	REE (kcal) Before	REE (kcal) After	Physical activity (METs) Before	Physical activity (METs) After
A11	86.10	85.40	27.20	27.00	18.40	21.10	105.50	105.00	2578.00	2494.00	32.85	29.00
A15	92.60	94.70	31.30	32.00	34.70	42.30	110.00	111.00	1497.00	1425.00	28.90	27.25
A17	101.50	103.90	31.30	32.10	29.40	30.80	111.00	112.50	2359.00	2871.00	33.35	34.24
A19	93.70	91.80	31.30	30.70	30.30	28.00	110.00	114.00	2354.00	1880.00	28.80	28.27
A5	78.10	75.40	33.80	32.60	32.60	35.10	111.00	107.00	1623.00	1545.00	35.55	32.10
A9	87.50	89.90	27.00	27.70	19.20	30.40	102.00	100.50	1632.00	2076.00	31.77	32.14
B11	132.00	127.70	41.00	39.40	47.00	48.50	132.00	131.00	2374.00	1631.00	29.30	26.50
B12	94.20	88.50	32.20	30.30	27.20	25.00	110.00	104.00	1885.00	1908.00	26.30	32.40
B19	80.40	77.80	25.40	24.60	16.00	13.00	99.00	95.00	1874.00	2200.00	35.70	26.07
B25	103.10	99.30	34.80	33.60	34.60	30.10	115.00	113.00	1837.00	1838.00	33.25	34.30
B3	77.10	75.00	26.40	25.60	13.80	15.80	96.00	93.00	1522.00	1671.00	23.00	47.40
B7	115.20	109.60	33.30	31.70	31.20	32.90	125.00	123.00	2490.00	2637.00	27.90	27.90
B8	82.00	80.30	28.40	27.80	16.40	19.30	99.00	98.00	1688.00	1615.00	24.70	37.49
C1	85.70	85.70	33.50	33.47	35.70	35.20	120.00	113.00	1744.00	1634.00	34.70	27.70
C12	116.90	118.10	35.30	35.70	31.20	37.60	124.00	127.50	1790.00	2918.00	28.35	30.86
C2	73.50	74.20	24.00	24.00	14.20	14.60	94.00	97.00	1587.00	1476.00	32.45	28.95
C21	97.70	96.10	31.20	30.70	27.70	26.40	115.00	115.00	2198.00	2041.00	37.10	32.25
C3	89.40	89.80	30.90	31.10	19.40	22.70	110.00	108.00	2126.00	1927.00	34.90	34.05
C6	101.00	97.70	34.50	33.40	35.20	33.90	120.00	118.00	2100.00	2725.00	39.90	35.30
D11	80.40	79.20	25.10	24.70	17.00	18.40	99.00	94.00	1565.00	1650.00	28.44	32.25
D19	101.50	98.50	35.10	34.10	44.10	46.40	109.00	104.00	2071.00	1726.00	31.34	26.50
D2	74.60	72.00	34.10	32.90	27.10	25.70	106.00	102.50	1649.00	1523.00	31.17	32.80
D21	93.10	95.00	29.40	30.00	23.00	25.90	113.00	116.00	2145.00	1752.00	28.05	29.45
D22	96.60	97.00	28.80	28.95	23.50	25.20	111.00	110.00	1858.00	2113.00	25.60	27.07
D3	84.80	85.50	31.90	31.40	24.00	26.00	105.00	104.00	1983.00	1640.00	31.90	31.05
D5	83.90	82.80	27.40	27.00	17.70	17.10	102.00	100.00	1561.00	1213.00	35.20	37.65

BMI: body mass index; REE: resting energy expenditure; MET: metabolic equivalent of tasks

EXCLI Journal 2020;19:241-250 – ISSN 1611-2156
Received: November 30, 2019, accepted: February 24, 2020, published: March 02, 2020

Supplementary Table 1 (cont.): Raw data of the FBS, insulin and lipid profile in each group before and after intervention. The below raw data are related to Table 1 and 2 in the main text.

Code	Insulin (mU/L) Before	Insulin (mU/L) After	FBS (mg/dL) Before	FBS (mg/dL) After	TG (mg/dL) Before	TG (mg/dL) After	Cholesterol (mg/dL) Before	Cholesterol (mg/dL) After	HDL (mg/dL) Before	HDL (mg/dL) After	LDL (mg/dL) Before	LDL (mg/dL) After
A11	10.00	7.80	151.00	134.00	314.00	263.00	112.00	136.00	22.00	20.00	42.00	63.00
A15	14.50	8.30	135.00	165.00	148.00	107.00	207.00	164.00	43.00	34.00	133.00	109.00
A17	6.90	10.90	107.00	124.00	268.00	162.00	156.00	137.00	39.00	34.00	63.00	71.00
A19	13.60	9.80	139.00	146.00	155.00	133.00	116.00	123.00	37.00	32.00	47.00	64.00
A5	8.80	11.00	278.00	269.00	133.00	155.00	176.00	171.00	46.00	45.00	100.00	99.00
A9	11.30	7.00	309.00	238.00	479.00	1407.00	227.00	321.00	26.00	21.00	113.00	69.00
B11	23.20	18.40	99.00	97.00	109.00	128.00	220.00	179.00	41.00	33.00	155.00	120.00
B12	12.00	4.50	132.00	113.00	184.00	148.00	193.00	164.00	33.00	23.00	126.00	111.00
B19	7.90	6.10	106.00	129.00	144.00	127.00	194.00	162.00	38.00	29.00	128.00	108.00
B25	18.70	14.70	87.00	100.00	99.00	141.00	112.00	113.00	33.00	27.00	56.00	58.00
B3	10.50	5.30	159.00	144.00	83.00	96.00	144.00	154.00	27.00	32.00	100.00	102.00
B7	19.00	23.60	196.00	166.00	485.00	337.00	210.00	208.00	14.00	21.00	100.00	118.00
B8	10.80	8.70	198.00	195.00	169.00	199.00	144.00	172.00	29.00	34.00	80.00	98.00
C1	14.40	17.30	140.00	122.00	111.00	153.00	149.00	178.00	48.00	54.00	71.00	94.00
C12	14.40	13.40	115.00	118.00	178.00	365.00	158.00	156.00	25.00	21.00	98.00	62.00
C2	8.90	9.60	197.00	199.00	120.00	139.00	133.00	181.00	27.00	34.00	74.00	119.00
C21	7.20	5.80	94.00	124.00	308.00	178.00	155.00	143.00	27.00	27.00	65.00	80.00
C3	22.70	13.70	158.00	178.00	300.00	259.00	173.00	210.00	28.00	39.00	69.00	119.00
C6	7.40	22.40	188.00	322.00	348.00	439.00	160.00	172.00	22.00	17.00	62.00	46.00
D11	9.10	4.70	113.00	97.00	95.00	82.00	173.00	150.00	52.00	41.00	98.00	93.00
D19	12.20	5.70	124.00	129.00	100.00	122.00	115.00	119.00	36.00	31.00	59.00	64.00
D2	11.70	8.10	103.00	124.00	71.00	79.00	109.00	136.00	44.00	50.00	45.00	70.00
D21	5.40	6.00	268.00	238.00	164.00	192.00	177.00	210.00	39.00	40.00	107.00	132.00
D22	12.70	11.90	115.00	138.00	296.00	355.00	153.00	182.00	28.00	27.00	64.00	76.00
D3	9.60	7.50	109.00	104.00	167.00	196.00	140.00	138.00	29.00	30.00	71.00	66.00
D5	8.60	11.00	106.00	119.00	122.00	147.00	182.00	189.00	42.00	41.00	114.00	116.00

FBS: fasting blood glucose; TG: triglyceride; LDL: Low-density lipoprotein; HDL: High-density lipoprotein

EXCLI Journal 2020;19:241-250 – ISSN 1611-2156
Received: November 30, 2019, accepted: February 24, 2020, published: March 02, 2020

Supplementary Table 1 (cont.): Raw data of the age, sex and liver function tests and fibroscan in each group before and after intervention. The below raw data are related to Table 1 and 2 in the main text.

Code	AST (U/L) Before	AST (U/L) After	ALT (U/L) Before	ALT (U/L) After	GGT (U/L) Before	GGT (U/L) After	Fibroscore (KPa) Before	Steatosis score (CAP) Before	Sex (male/ female)	Age (year)
A11	20.00	24.00	18.00	18.00	45.00	38.00	5.00	300.00	male	41.00
A15	17.00	13.00	10.00	8.00	26.00	28.00	5.00	340.00	famale	47.00
A17	18.00	18.00	19.00	15.00	24.00	22.00	4.50	270.00	male	36.00
A19	17.00	15.00	18.00	10.00	21.00	18.00	4.00	325.00	male	49.00
A5	34.00	19.00	28.00	20.00	46.00	37.00	7.00	300.00	famale	47.00
A9	22.00	25.00	27.00	50.00	50.00	65.00	5.30	285.00	male	40.00
B11	26.00	29.00	11.00	19.00	28.00	27.00	13.00	399.00	male	49.00
B12	39.00	21.00	31.00	13.00	46.00	37.00	8.00	350.00	male	49.00
B19	25.00	21.00	19.00	11.00	30.00	22.00	4.50	305.00	male	39.00
B25	35.00	27.00	46.00	28.00	30.00	28.00	7.00	360.00	male	39.00
B3	40.00	24.00	45.00	25.00	42.00	29.00	5.00	325.00	male	40.00
B7	31.00	29.00	26.00	26.00	64.00	65.00	10.00	370.00	male	46.00
B8	21.00	24.00	23.00	26.00	35.00	36.00	7.00	300.00	male	45.00
C1	21.00	17.00	19.00	16.00	23.00	13.00	6.00	320.00	famale	46.00
C12	26.00	28.00	19.00	19.00	22.00	18.00	8.50	365.00	male	43.00
C2	23.00	20.00	18.00	19.00	28.00	27.00	5.50	300.00	male	51.00
C21	21.00	19.00	15.00	9.00	25.00	20.00	5.30	303.00	male	53.00
C3	35.00	31.00	34.00	32.00	81.00	54.00	11.50	270.00	male	43.00
C6	20.00	30.00	21.00	29.00	31.00	22.00	6.80	356.00	male	49.00
D11	23.00	17.00	18.00	12.00	15.00	13.00	4.50	260.00	male	46.00
D19	20.00	18.00	15.00	13.00	22.00	28.00	5.00	383.00	famale	43.00
D2	24.00	25.00	20.00	24.00	19.00	22.00	5.50	340.00	famale	50.00
D21	13.00	32.00	9.00	17.00	38.00	188.00	5.00	300.00	male	48.00
D22	23.00	31.00	24.00	23.00	54.00	66.00	6.00	285.00	male	40.00
D3	26.00	32.00	16.00	20.00	34.00	33.00	8.50	318.00	male	42.00
D5	28.00	20.00	18.00	18.00	28.00	26.00	4.30	280.00	male	52.00

ALT: alanin aminotransferase; AST: aspartate aminotransferase; GGT: γ-glutamyl transferase

EXCLI Journal 2020;19:241-250 – ISSN 1611-2156
Received: November 30, 2019, accepted: February 24, 2020, published: March 02, 2020

Supplementary Table 2: Raw data of the log_{10} number per gram of feces of *Lactobacillus* in each group. The below raw data are related to Figure 2 in the main text.

groups	log_{10} number per gram													
	Before							After						
CFCA	7.54	6.43	5.45	8.54	9.34	8.54	7.45	7.89	7.64	5.63	8.56	9.56	8.45	8.76
CFPL	7.43	6.56	5.98	7.67	8.87	9.67		7.67	6.52	6.34	8.43	9.45	8.12	
CAPL	8.23	7.45	5.87	6.98	9.34	8.45		7.87	6.45	5.43	8.67	9.43	7.65	
PLPL	8.46	7.76	5.87	7.56	8.67	8.89		7.12	6.45	5.75	8.67	9.56	8.45	

CF: Chlorogenic acid; CA: caffeine; PL: placebo

Supplementary Table 2 (cont.): Raw data of the log_{10} number per gram of feces of *Bacteroides* in each group. The below raw data are related to Figure 2 in the main text.

groups	log_{10} number per gram													
	Before							After						
CFCA	6.5	5.8	7.44	6.4	5.12	6.34	6.45	5.98	5.56	6.98	6.12	6.23	6.45	6.24
CFPL	5.45	7.45	6.53	6.76	6.87	7.34		5.56	6.98	6.43	6.73	6.75	7.12	
CAPL	4.56	5.76	6.87	7.98	8.76	6.5		5.01	5.76	6.78	8.12	8.5	6.23	
PLPL	6.5	5.98	7.5	6.43	6.45	4.98		6.52	6.02	8.23	6.52	6.63	6.54	

CF: Chlorogenic acid; CA: caffeine; PL: placebo

Received: November 30, 2019, accepted: February 24, 2020, published: March 02, 2020

Supplementary Table 2 (cont.): Raw data of the log_{10} number per gram of feces of *Bifidobacterium* in each group. The below raw data are related to Figure 2 in the main text.

groups	log_{10} number per gram													
	Before							After						
CFCA	8.22	7.54	5.45	9.34	10.21	9.32	8.55	8.65	7.64	8.45	9.54	10.43	9.5	9.32
CFPL		7.43	6.13	8.55	9.98	10.12		8.46	6.52	6.34	9.36	10.32	9.43	
CAPL		8.23	6.32	7.45	10.34	9.46		8.54	7.43	5.43	9.42	11	8.88	
PLPL		8.46	6.54	8.45	9.86	9.96		8.12	7.44	5.75	9.76	10.43	9.35	

CF: Chlorogenic acid; CA: caffeine; PL: placebo
